# Precision Genome Engineering Through Cytidine Base Editing in Rapeseed (*Brassica napus*. L)

**DOI:** 10.3389/fgeed.2020.605768

**Published:** 2020-11-20

**Authors:** Limin Hu, Olalekan Amoo, Qianqian Liu, Shengli Cai, Miaoshan Zhu, Xiaoxiao Shen, Kaidi Yu, Yungu Zhai, Yang Yang, Lei Xu, Chuchuan Fan, Yongming Zhou

**Affiliations:** National Key Laboratory of Crop Genetic Improvement, Huazhong Agricultural University, Wuhan, China

**Keywords:** *Brassica napus*, base-editing, cytidine deaminase, gain-of-function mutation, crop improvement

## Abstract

Rapeseed is one of the world's most important sources of oilseed crops. Single nucleotide substitution is the basis of most genetic variation underpinning important agronomic traits. Therefore, genome-wide and target-specific base editing will greatly facilitate precision plant molecular breeding. In this study, four CBE systems (BnPBE, BnA3A-PBE, BnA3A1-PBE, and BnPBGE14) were modified to achieve cytidine base editing at five target genes in rapeseed. The results indicated that genome editing is achievable in three CBEs systems, among which BnA3A1-PBE had the highest base-editing efficiency (average 29.8% and up to 50.5%) compared to all previous CBEs reported in rapeseed. The editing efficiency of BnA3A1-PBE is ~8.0% and fourfold higher, than those of BnA3A-PBE (averaging 27.6%) and BnPBE (averaging 6.5%), respectively. Moreover, BnA3A1-PBE and BnA3A-PBE could significantly increase the proportion of both the homozygous and biallelic genotypes, and also broaden the editing window compared to BnPBE. The cytidine substitution which occurred at the target sites of both *BnaA06.RGA* and *BnaALS* were stably inherited and conferred expected gain-of-function phenotype in the T1 generation (i.e., dwarf phenotype or herbicide resistance for weed control, respectively). Moreover, new alleles or epialleles with expected phenotype were also produced, which served as an important resource for crop improvement. Thus, the improved CBE system in the present study, BnA3A1-PBE, represents a powerful base editor for both gene function studies and molecular breeding in rapeseed.

## Introduction

Rapeseed (*Brassica napus* L., AACC, 2n = 38) is one of the most important sources of oilseed crops in the world, accounting for ~16% of the entire global vegetable oil production (Woodfield et al., 2017). Achieving high yields through genetic improvements has always been the major goal in rapeseed production. The constant creation and use of novel genetic variation are important to both genetic research and plant trait improvement. In order to optimize the agronomic traits of crops, breeders applied various methods such as chemical compounds and irradiation to produce heritable mutations. However, these traditional techniques are not target-specific and require genome-scale screening, which is time-and-labor-consuming (Russell et al., 1958; Sega, 1984). As an allotetraploid species, rapeseed has a complicated genome in which most genes have several homologous copies (Chalhoub et al., 2014). Thus, obtaining mutations at all homologous copies is challenging by traditional mutagenesis. With the rapid progress in molecular biology, genome-editing technologies have proven to be a powerful tool to address this issue.

In recent years, CRISPR/Cas9 systems have been proven to be very efficient in improving agronomic traits, especially yield-related traits of rapeseed through genome editing (Braatz et al., 2017; Yang et al., 2018; Zhai et al., 2020). The traditional CRISPR/Cas9 system prefers to generate small insertions and deletions (indels) and is best suited to create knockout mutations. This makes the traditional CRISPR/Cas9 system ineffective when precise base substitutions are needed. However, many desired agronomic traits involve only single nucleotide variants within genes, such as the reported cytidine (C) to thymidine (T) replacement at particular sites of *BnaALS, BnaRGA*, and *BnaA3.IAA7* that conferred gain-of-function mutations with valuable benefits for agricultural applications in rapeseed (Liu et al., 2010; Li et al., 2015, 2019). Therefore, precise base editing has great potential for the production of desired alleles and trait improvement. Using this method, desirable traits can be introgressed into elite lines without compromise, and the resulting lines with targeted improvement will be utilized for practical production.

Recently, base editors, including cytidine base editors (CBEs) and adenine base editors (ABEs), enable precise base alterations in the genome without inducing DNA double-stranded breaks (DSBs) (Komor et al., 2016; Nishida et al., 2016; Gaudelli et al., 2017). CBEs using a Cas9 variant fused with cytidine deaminase have enabled C-to-T conversion without requiring DSBs formation and homology-directed repair in mammalian cells (Komor et al., 2016). Currently, the most commonly used CBE, named BE3, consists of the rat cytidine deaminase APOBEC1 (rAPOBEC1) and uracil DNA glycosylase inhibitor (UGI) fused to Cas9 nickase (nCas9) (Li et al., 2017; Lu and Zhu, 2017; Ren et al., 2017; Zong et al., 2017). The BE3 system typically allows C-T substitution within a small editing window from C4 to C8 of the protospacer (Komor et al., 2016; Li et al., 2017). Several studies have reported the successful applications of CBE in several crop species including rice, maize, wheat, tomato, cotton, and rapeseed (Li et al., 2017; Zong et al., 2017, 2018; Qin et al., 2019; Wu et al., 2020). In addition, many other base editing systems have been developed in plants to improve gene editing accuracy and efficiency. A *Petromyzon marinus* cytidine deaminase (PmCDA1)-based CBE has resulted in efficient editing in rice and tomato (Shimatani et al., 2017). Moreover, a separate study has shown that PmCDA1 has higher base editing activity than rAPOBEC1 in rice (Tang et al., 2018). Zong et al. (2018) further improved CBEs by using the more effective human APOBEC3A (named A3A-PBE) which worked efficiently in wheat, rice and potato with a 17-nucleotide editing window, independent of sequence context.

During the preparation of our manuscript, Wu et al. (2020) reported the successful application of cytosine base-editing in rapeseed using rat cytidine deaminase APOBEC1. The editing efficiency was 1.8%, which is relatively lower when compared to other crops, and only one copy of *BnaALS* gene was edited (Wu et al., 2020). Cheng et al. (2020) successfully used A3A-PBE system to target *ALS, RGA*, and *IAA7* genes with an averaging editing efficiency of 23.6%, which also needs further improvement in the editing efficiency. In addition, they provided very limited information on the editing feature of the A3A-PBE system (Cheng et al., 2020). Therefore, further studies are required to establish more effective CBE systems in rapeseed based on the commonly used cytidine deaminases like rAPOBEC1, PmCDA1, and APOBEC3A.

In this study, we modified four CBE systems to achieve cytidine base editing at different genome sites in rapeseed. Five important genes with well-known functions, including *BnaCLV3, BnaRGA, BnaA3.IAA7, BnaDA1*, and *BnaALS*, were selected for precise base editing to improve agronomic traits in rapeseed (Liu et al., 2010; Li et al., 2015, 2019; Wang et al., 2017; Yang et al., 2018). Our results indicated that BnA3A1-PBE represents the best CBE editor in rapeseed at present, with the highest base-editing efficiency (up to 50.5%) and higher proportion of both homozygous and biallelic genotypes. The cytidine substitution that occurred at the target sites of *BnaRGA* and *BnaALS* were stably inherited and conferred expected phenotype in the T1 generation, indicating its powerful application prospect in rapeseed improvement.

## Methods

### Vector Construction

To construct BnPBE and BnA3A-PBE vectors, cytidine deaminase (rAPOBEC1 or APOBEC3A), nCas9 and UGI units were amplified from pnCas9-PBE or A3A-PBE template plasmid (Zong et al., 2017), while the 35S promoter and ccdB units were amplified from PYLCRISPRCas9P35s-H (Ma et al., 2015). The resulting polymerase chain reaction (PCR) products were inserted into the *Pme*I/*BamH*I sites of binary vector PYLCRISPRCas9P35s-H through a Pro Ligation-Free Cloning Kit (Applied Biological Materials Inc, Canda, Cat.No.E086/E087). Furthermore, the cereal plant APOBEC3A sequences were codon-optimized for dicotyledon plant and synthesized commercially (Nanjing, China, GenScript) to create BnA3A1-PBE. The multiple sgRNA constructs were generated following a previous protocol used in combining sgRNAs to PYLCRISPRCas9P35s-H (Yang et al., 2018). Then, the multiple sgRNAs were amplified from the generating vector and the resulting PCR product was inserted into the *Asc*I sites of BnPBE, BnA3A-PBE and BnA3A1-PBE, using the Pro Ligation-Free Cloning Kit. To construct BnPBGE14 vector, nCas9 and PmCDA1 (Shimatani et al., 2017) were codon-optimized for dicotyledon plant and then synthesized commercially to replace Cas9 in PYLCRISPRCas9P35s-H. The multiple target sequences were synthesized and ligated to the *Bsa*I sites of PYLCRISPRCas9P35s-H. Primers used for vector construction are listed in Supplementary Table 1.

### *Agrobacterium*-Mediated Rapeseed Transformation

Following verification of the fused constructs via sequencing, the CBE expressing binary vectors were transformed into an elite cultivar (J9707) via the *Agrobacterium tumefaciens*-mediated hypocotyl method (Zhou and Fowke, 2002). Hygromycinselection (25 mg/L) was used to screen the transgenic plants.

### On-Target Mutation Analysis by Targeted Deep Sequencing

Genomic DNA was extracted from the T_0_ transgenic and wild type rapeseed plants using the CTAB method. The positive transgenic plants were screened by PCR using the specific primer pairs PB-L/PB-R (Supplementary Table 1). Then, the targeted mutations were determined in transgenic plants using the high-throughput tracking of mutations (Hi-TOM) platform (Liu et al., 2019). The sequencing analysis was conducted following the approach previously described by Zhai et al. (2020). The targets specific primer sets are listed in Supplementary Table 1.

### Off-Target Analysis

Putative off-target sites, which contained 2–4-nucleotide mismatches relative to the *BnaCLV3* and *BnaRGA* target sites, were identified using Cas-OFFinder and CRISPR-P software (Bae et al., 2014; Liu et al., 2017). These potential off-target sites were detected in all edited T_0_ transgenic plants using targeted deep sequencing. For each target gene, mixed genomic DNA from all T_0_ editing plants was used as the template, and DNA of wild type plant was included as a control. All PCR products were purified and mixed in equal amounts (50 ng for each) as one sample. The DNA library construction, sequencing using the Illumina HiSeq 3000 system and data analysis were conducted according to the methods previously described by Yang et al. (2018). The independent sequence reads of each off-target site were aligned to the genomic wild type sequence, which covered each off-target site as a reference.

### Herbicide Resistance Test

The T_1_ mutants and wild type plants grown in the greenhouse (23°C, 16 h light/20°C, 8 h dark) were treated with commercial sulfonylureas at 1, 2, and 4 times field-recommended concentration (200, 400, and 800 mg/L). Representative pictures were taken 3 weeks after treatment.

## Results and Discussion

### Design of Four CBE Systems and sgRNA Expression Cassettes

**Figure 1:**
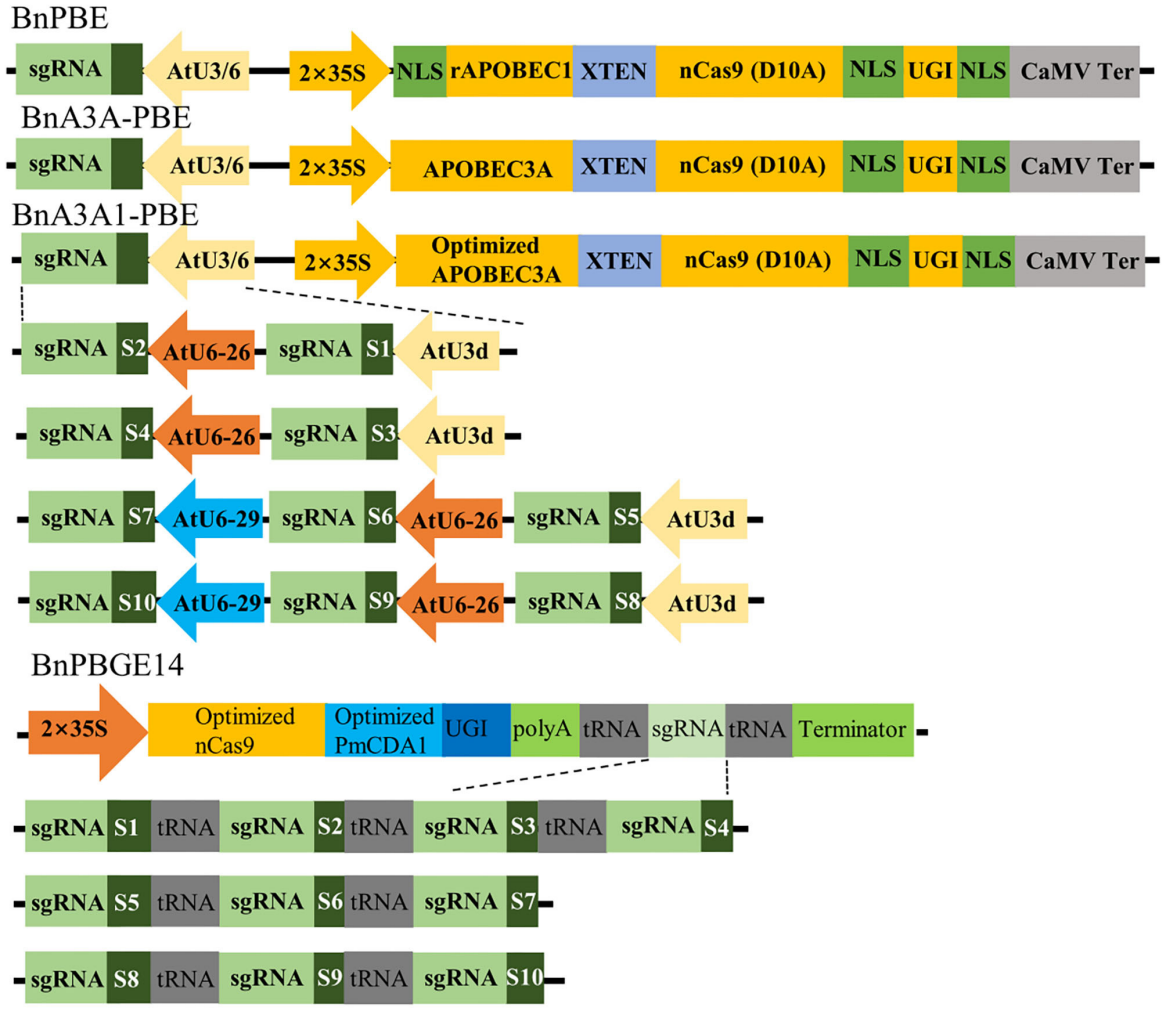
Schematic representation of four CBE systems and 10 sgRNAs used in this study. The main difference among BnPBE, BnA3A-PBE, BnA3A1-PBE, and BnPBGE1 is the sequence of cytidine deaminase. Ten sgRNAs were tested in each CBE system to compare the editing efficiency of the different systems.

In this study, we adopted the base editor units (cytidine deaminase, nCas9 and UGI) from the PBE and A3A-PBE plasmid (Zong et al., 2018) to replace Cas9 in the pYLCRISPR/Cas9P35S-H binary vector (Yang et al., 2018), leading to the BnPBE and BnA3A-PBE systems, respectively. A codon-optimized APOBEC3A for *Brassica* plants was synthesized to optimize A3A-PBE, resulting in the creation of BnA3A1-PBE (Figure 1). PmCDA1 and nCas9 sequences (Shimatani et al., 2017) were codon-optimized for *Brassica* plants and synthesized to replace Cas9 in the pYLCRISPR/Cas9P35S-H binary vector, leading to the BnPBGE14 system (Figure 1). Thus, four CBE systems were modified to test cytidine base editing in *B. napus*. Three of them, including BnPBE, BnA3A-PBE, and BnA3A1-PBE, used the 35S promoter and the AtU3/AtU6 promoters to express the base editor unit and sgRNAs, respectively; while, BnPBGE14 use a 35S promoter to express both the base editor unit and sgRNAs in one ORF, in which multiple sgRNAs were further released using the tRNA-processing system-based strategy (Figure 1).

To investigate the feasibility and efficacy of these CBE systems in rapeseed, we designed 10 sgRNAs for five endogenous genes: sgRNA1 (S1) and S2 for *BnaCLV3*, S3 for *BnaRGA*, S4 for *BnaA3.IAA*, S5 to S7 for *BnaDA1*, and S8 to S10 for *BnaALS*. Then, four constructs for each CBE system were generated and introduced separately into the rapeseed variety J9707 through *Agrobacterium*-mediated transformation. An average of 78 independent T_0_ transgenic lines were generated for each of the 15 CRISPR constructs (Table 1, Supplementary Table 2).

**Table 1:** Detail information of the numbers of T_0_ plants with different mutation types.

**Name of vector**	**Target gene**	**sgRNA**	**No. of plants examined**	**No. of plants with INDEL**	**No. of plants with C-A/G base editing**	**No. of T**_**0**_ **plants with C-T base editing**				**The ratio of edited T_**0**_ plants**
						**HE**	**HO**	**Bi-allelic**	**Chimeric**	
BnPBE-1	*BnaA04.CLV3*	sgRNA1	107	0 (0.0%)	0 (0.0%)	9	0	0	0	7/107 (6.5%)
		sgRNA2								
	*BnaC04.CLV3*	sgRNA1	112	0 (0.0%)	0 (0.0%)	2	1	0	0	3/112 (2.7%)
		sgRNA2								
BnA3A-PBE1	*BnaA04.CLV3*	sgRNA1	81	0 (0.0%)	7 (9.1%)	21	9	12	5	47/81 (58.0%)
		sgRNA2								
	*BnaC04.CLV3*	sgRNA1	77	3 (3.9%)	5 (6.5%)	11	11	20	2	44/77 (57.1%)
		sgRNA2								
BnA3A1-PBE1	*BnaA04.CLV3*	sgRNA1	86	1 (1.2%)	7 (8.1%)	15	9	12	6	42/86 (48.8%)
		sgRNA2								
	*BnaC04.CLV3*	sgRNA1	84	2 (2.4%)	2 (2.4%)	9	14	8	3	34/84 (40.5%)
		sgRNA2								
BnPBE-2	*BnaA06.RGA*	sgRNA3	87	0 (0.0%)	0 (0.0%)	21	1	6	0	28/87 (32.2%)
BnA3A-PBE2	*BnaA06.RGA*	sgRNA3	80	4 (5.0%)	2 (2.5%)	7	12	11	5	35/80 (43.8%)
BnA3A1-PBE2	*BnaA06.RGA*	sgRNA3	95	11 (11.6%)	4 (4.2%)	8	10	24	6	48/95 (50.5%)
BnPBE-2	*BnaA03.IAA7*	sgRNA4	86	0 (0.0%)	0 (0.0%)	1	0	0	0	1/86 (1.2%)
BnA3A-PBE2	*BnaA03.IAA7*	sgRNA4	83	1 (1.2%)	0 (0.0%)	10	0	3	1	14/83 (16.8%)
BnA3A1-PBE2	*BnaA03.IAA7*	sgRNA4	95	7 (7.4%)	2 (2.1%)	13	2	5	1	21/95 (22.1%)
BnPBE-3	*BnaA06DA1*	sgRNA5	93	0 (0.0%)	0 (0.0%)	0	0	0	0	0 (0.0%)
	*BnaC05DA1*	sgRNA6	93	0 (0.0%)	0 (0.0%)	0	0	0	0	0 (0.0%)
	*BnaA08.DA1*	sgRNA7	93	0 (0.0%)	0 (0.0%)	0	0	0	0	0 (0.0%)
	*BnaC08.DA1*	sgRNA5	93	0 (0.0%)	0 (0.0%)	0	0	0	0	0 (0.0%)
BnA3A-PBE3	*BnaA06.DA1*	sgRNA5	79	3 (3.8%)	0 (0.0%)	4	1	4	0	9/79 (11.4%)
	*BnaC05.DA1*	sgRNA6	79	4 (5.1%)	1 (1.3%)	4	1	0	1	6/79 (7.6%)
	*BnaA08.DA1*	sgRNA7	83	1 (1.2%)	0 (0.0%)	2	0	0	2	4/83 (4.8%)
	*BnC08.DA1*	sgRNA5	83	1 (1.2%)	0 (0.0%)	9	2	0	0	11/83 (13.3%)
BnA3A1-PBE3	*BnaA06.DA1*	sgRNA5	49	1 (2.0%)	0 (0.0%)	3	1	2	0	6/49 (12.2%)
	*BnaC05.DA1*	sgRNA6	49	2 (4.1%)	0 (0.0%)	3	1	1	0	5/49 (10.2%)
	*BnaA08.DA1*	sgRNA7	49	1 (2.0%)	0 (0.0%)	2	3	1	0	6/49 (12.2%)
	*BnaC08.DA1*	sgRNA5	49	1 (2.0%)	0 (0.0%)	7	0	1	0	8/49 (16.3%)
BnPBE-4	*BnaALS3*	sgRNA8	159	0 (0.0%)	0 (0.0%)	2	0	0	0	2/159 (1.3%)
	*BnaALS3*	sgRNA10	159	0 (0.0%)	0 (0.0%)	8	0	2	0	10/159 (6.3%)
	*BnaALS1*	sgRNA9	159	0 (0.0%)	0 (0.0%)	8	1	0	0	9/159 (5.7%)
	*BnaALS1*	sgRNA10	159	2 (1.3%)	0 (0.0%)	9	0	0	2	11/159 (7.0%)
BnA3A-PBE4	*BnaALS3*	sgRNA8	107	1 (0.9%)	0 (0.0%)	9	0	0	1	10/107 (9.3%)
	*BnaALS3*	sgRNA10	106	7 (6.6%)	0 (0.0%)	22	11	9	2	44/106 (41.5%)
	*BnaALS1*	sgRNA9	108	3 (2.8%)	1 (0.9%)	25	15	0	0	40/108 (37.0%)
	*BnaALS1*	sgRNA10	108	10 (9.3%)	1 (0.9%)	21	13	11	2	47/108 (43.5%)
BnA3A1-PBE4	*BnaALS3*	sgRNA8	80	0 (0.0%)	0 (0.0%)	14	4	0	0	18/80 (22.5%)
	*BnaALS3*	sgRNA10	80	0 (0.0%)	0 (0.0%)	18	6	6	0	30/80 (37.5%)
	*BnaALS1*	sgRNA9	81	0 (0.0%)	0 (0.0%)	18	9	3	0	30/81 (37.0%)
	*BnaALS1*	sgRNA10	81	1 (1.2%)	0 (0.0%)	16	6	10	1	33/81 (40.7%)

### Detection of the Base-Editing of Different CBE Systems

**Figure 2:**
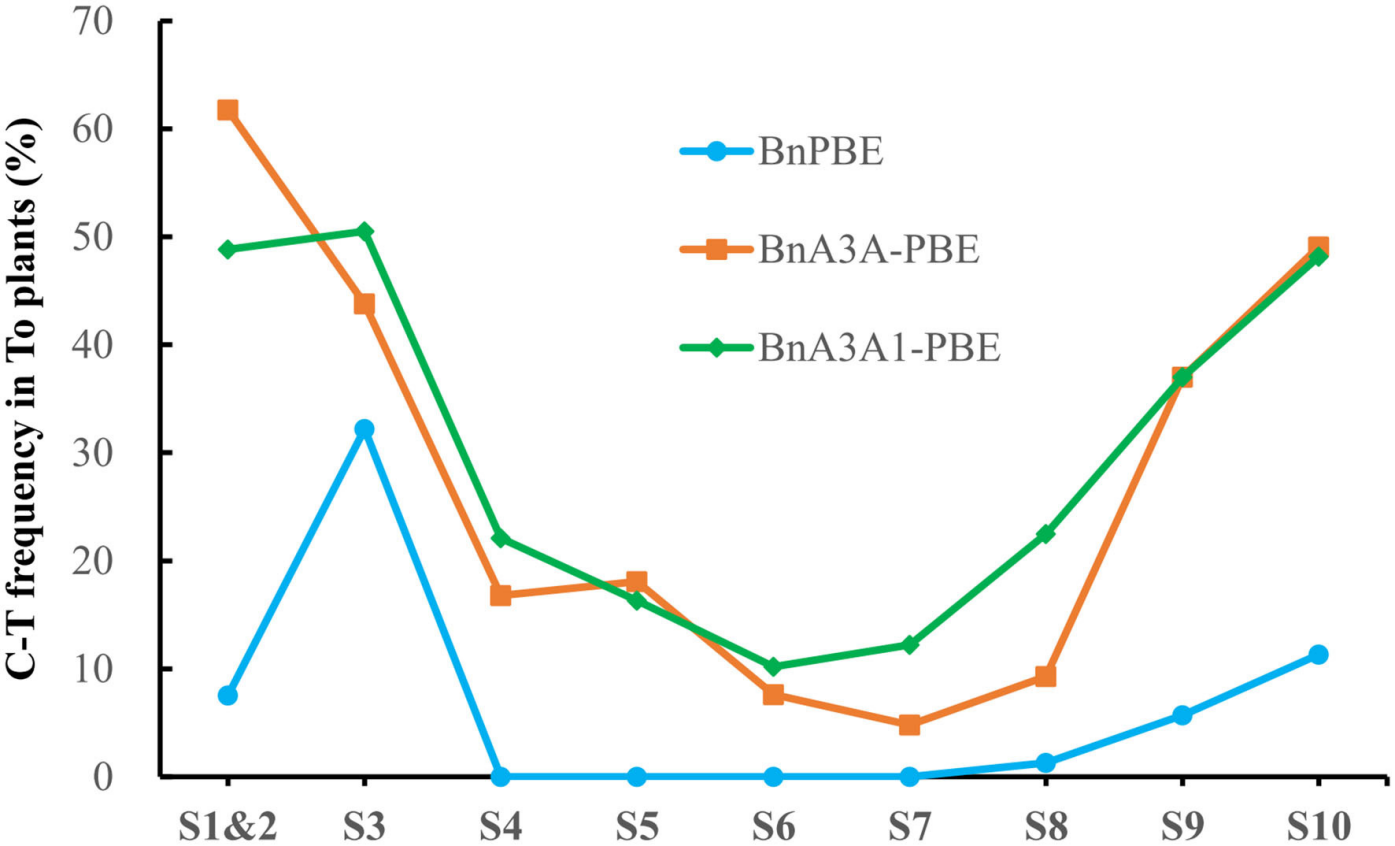
Frequencies of C-to-T conversion using three active CBE systems at 10 sgRNAs in T_0_ plants.

Base-editing of the generated plants was assessed by Hi-TOM through sequencing of the sgRNA target sites (Liu et al., 2019). The observed mutations at the S1 and S2 sites in this study were considered as one due to the overlapping of the sgRNA sequences. It showed that three of CBEs (BnPBE, BnA3A-PBE, and BnA3A1-PBE) were active at all sgRNAs, except BnPBE showed no editing at the S4-to-S7 sites (Figure 2). And the BnPBGE14 was inactive at all sgRNAs (Supplementary Table 2). BnA3A1-PBE had the highest base-editing efficiency, with an average editing efficiency of 29.8%, which is ~8.0% and fourfold higher than those of BnA3A-PBE (averaging 27.6%) and BnPBE (averaging 6.5%), respectively (Figure 2; Table 1). The C-to-T substitution efficiencies reached up to 50.5% in BnA3A1-PBE (Figure 2; Table 1), which is comparable with the efficiency in other crops (Qin et al., 2019). Recently, there were two reports about the successful application of different CBE systems in rapeseed [i.e., a PBE system (1.8% editing efficiency) reported by Wu et al. (2020) and an A3A-PBE system (averaging 23.6% editing efficiency) reported by Cheng et al. (2020)]. The performance of BnA3A1-PBE was much better than these two reported CBE systems, and thus represents the best CBE editor in rapeseed at present.

The overall base-editing efficiencies of the three CBE systems showed a similar trend at all sgRNAs [i.e., a higher editing efficiency at S1–S3, S9, and S10 and a lower editing efficiency at S4–S7 (Figure 2)]. In accordance with previous results, the difference in editing efficiency at these sgRNAs might be due to their nucleotide composition, GC content or promoter activities. Based on the fact that base editing with rAPOBEC1 is limited to a narrow deamination window and is inefficient in the GC contexts (Komor et al., 2016; Zong et al., 2018), this might be the reason why BnPBE has no editing activity at the S4–S7 sites.

### Comparison of the Mutation Features in Different CBE Systems

**Figure 3:**
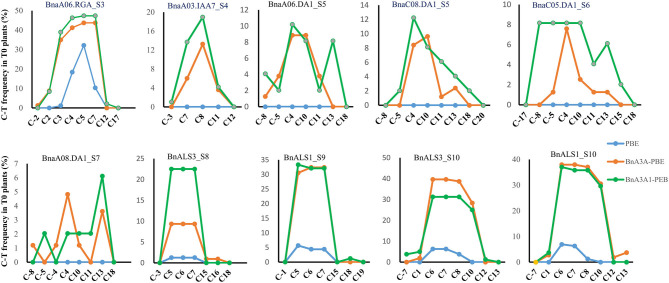
Frequencies of single C-to-T conversions by BnPBE, BnA3A-PBE, and BnA3A1-PBE at 10 target sites in T_0_ plants. Substitution efficiency was calculated by the ratio of the edited plants in the total transgenic positive plants.

Analyses of the base-editing efficiencies at every protospacer position across different sgRNAs revealed that the deamination window for BnA3A1-PBE spanned 17 nucleotides from protospacer positions 2–18, compared with 2–16 for BnA3A-PBE, and 3–8 for BnPBE (Figure 3), which is consistent with previous reports (Zong et al., 2018). Furthermore, we found that the on-target editing products were different among the active three CBE systems: BnA3A-PBE and BnA3A1-PBE preferred to substitute more C into T simultaneously compared with BnPBE. For example, at the S10 site, BnPBE created four types of mutations with one to three substitutions (C6, C6C7 or C6C7C8), while BnA3A-PBE and BnA3A1-PBE created six types of mutations, where simultaneous editing of three or four Cs (C6C7C8 and C6C7C8C10) occurred more frequently (Supplementary Table 3). These results suggested that there was an obvious difference in the main mutation genotypes between rat-APOBEC1-based BnPBE and human-APOBEC3A-based BnA3A-PBE or BnA3A1-PBE. Thus, BnA3A-PBE and BnA3A1-PBE could increase the production of novel alleles with diverse genetic variations because of their broad editing window. Whereas, BnPBE could reduce the possibility of introducing undesired mutations at specific sites because of the narrow editing window. However, BnPBE has a lower editing efficiency than BnA3A-PBE and BnA3A1-PBE (Figures 2, 3). Therefore, it is critical to fully understand the characteristics of these editing systems for better utilization.

**Figure 4:**
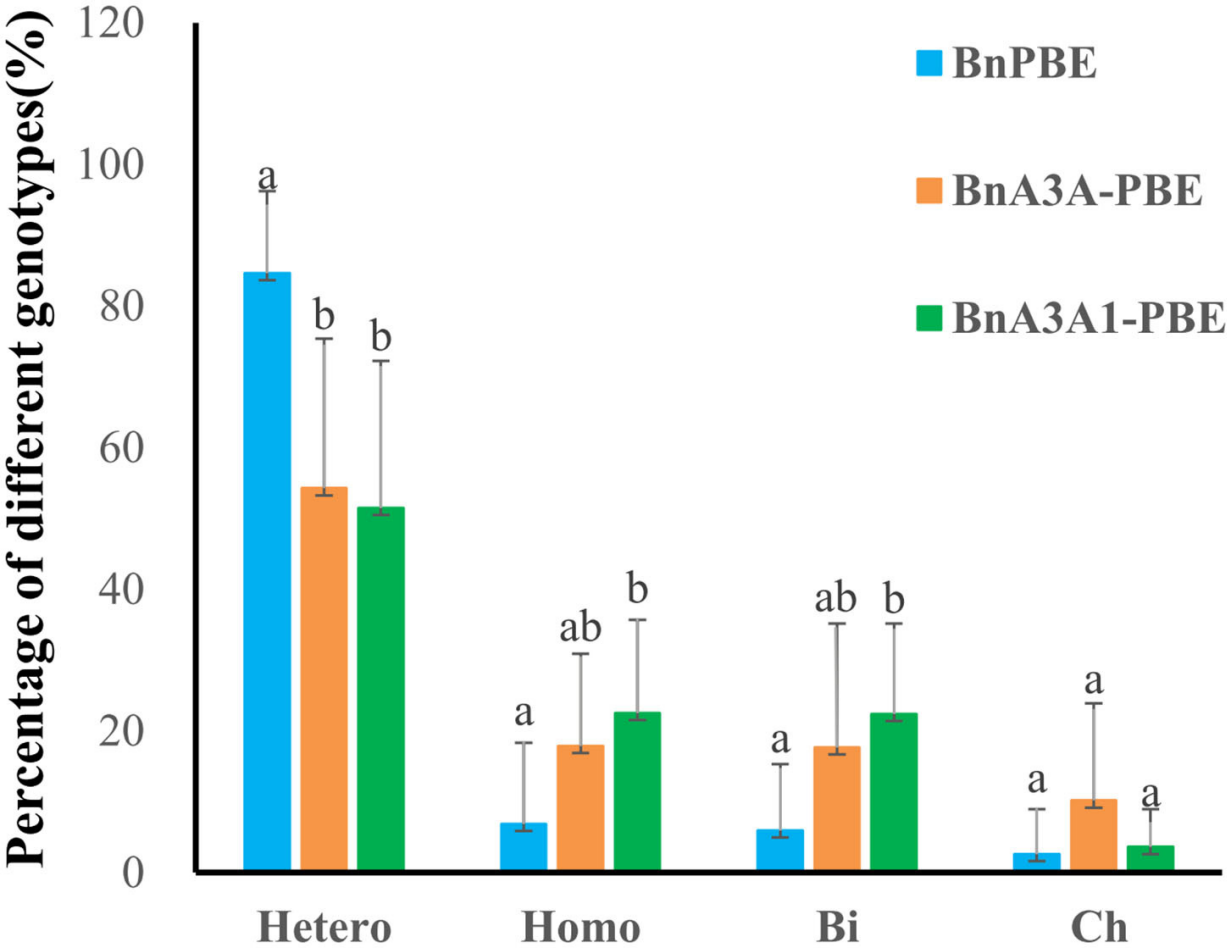
Percentage of four genotypes of base mutations in 10 targets using three CBE systems. Uppercase letters indicate a significant difference at the 0.05 probability level among the three CBE systems based on a multiple comparison test. Hetero, heterozygous; Homo, homozygous; Bi, biallelic; Ch, chimerism.

By analyzing the ratio of different mutation genotypes in base-edited plants, the three active CBE systems produced mutants with a similar trend [i.e, heterozygous (Hetero) > homozygous (Homo) > biallelic (Bi) > chimerism (Ch) (Figure 4)]. Compared with BnPBE, BnA3A-PBE, and BnA3A1-PBE could significantly reduce the proportion of heterozygous genotypes and increase the proportion of both homozygous and biallelic genotypes (Figure 4).

We compared the base-editing efficiencies of these sgRNA sites that could target both the A and C subgenomes of rapeseed in this study. The data showed that all the CBEs had uniform editing rate between the two subgenomes for six sgRNAs, whereas an obvious bias of base editing at S8 and S9 was observed in the C-subgenome (26.7% on average) compared to that in the A-subgenome (11.0% on average). More than 80% of base editing in BnPBE occurred at only C subgenomes, whereas more than 56.7 and 61.7% of base editing occurred simultaneously at both subgenomes for BnA3A-PBE and BnA3A1-PBE, respectively (Table 1).

The overall unexpected nucleotide changes and indels in the putative editing window occurred with much lower frequencies than C-to-T base editing, and BnA3A-PBE and BnA3A1-PBE yielded relatively higher frequencies of these undesired edits than that those observed in BnPBE (Table 1). This showed that the frequency of undesired edits was positively correlated with the editing efficiency of CBE systems.

### Off-Target Activity of the CBE Systems in T_0_ Transgenic Rapeseed Plants

To detect any potential off-target effects of the CBE systems reported here, we selected the target sites with the highest editing efficiency corresponding to the three active CBE systems to detect the off-target efficiency. There were 23 and four potential off-target sites identified for *BnaCLV3* and *BnaRGA* sites, respectively, with up to 4-nucleotide mismatches (Bae et al., 2014; Liu et al., 2017). High-throughput sequencing of the PCR products of these potential off-target sites revealed that no significant difference was observed in the off-target ratio between the base-edited and wild-type plants (Supplementary Table 4). These results revealed that the three active CBE systems have a high specificity for targeted mutagenesis in rapeseed, which is consistent with previous reports in animals and plants (Kim et al., 2017; Qin et al., 2019; Cheng et al., 2020).

### The Base Editing of the *BnaRGA* Gene Produced an Expected Dwarf Phenotype

**Figure 5:**
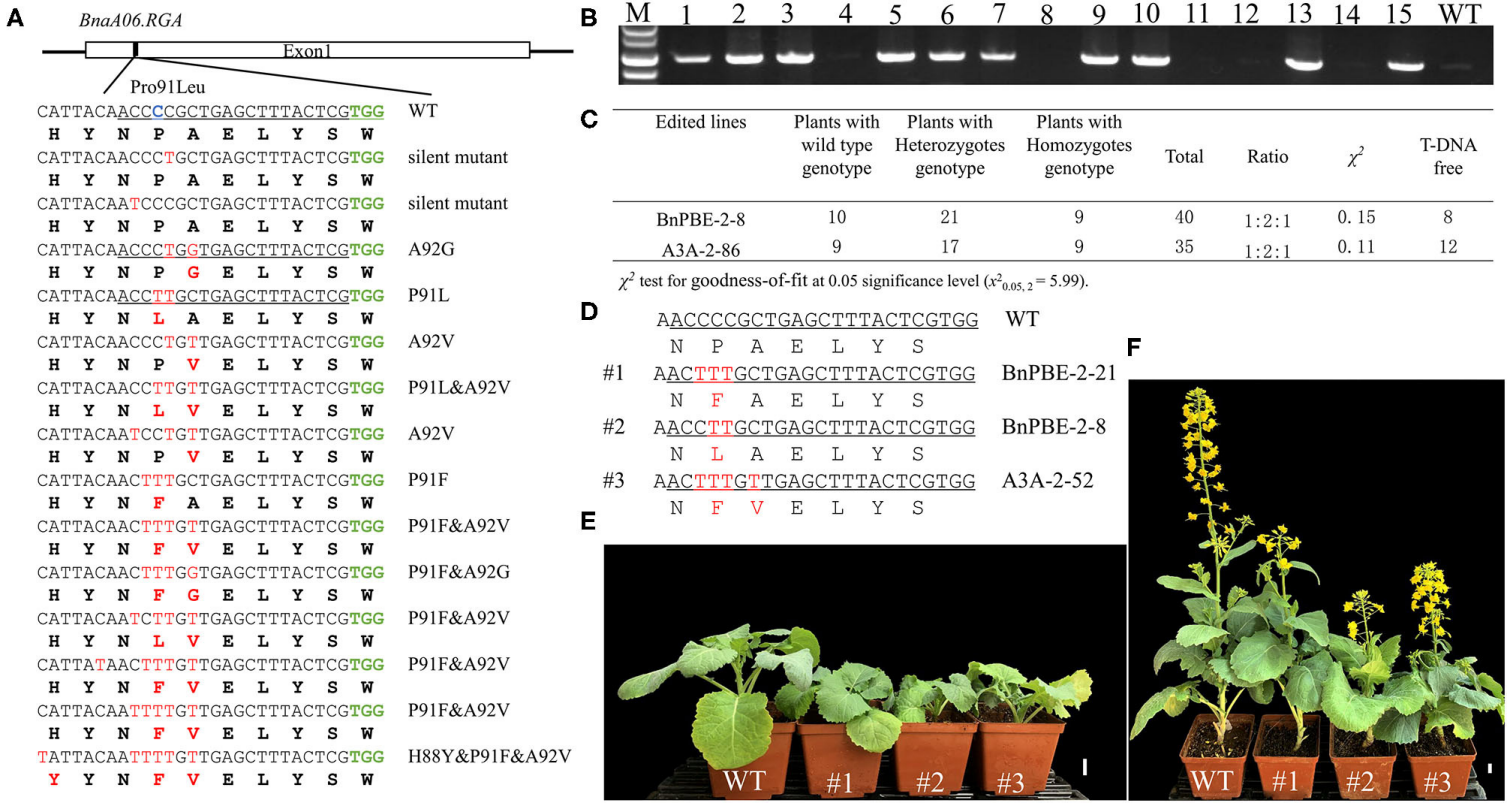
P91 substitution in *BnaA06.RGA* T_0_ mutants confer a dwarf phenotype. **(A)** Diverse editing events at the P91 site of *BnaA06.RGA*. Target sequence, expected substitution, PAM sequence and DNA or protein modifications are indicated by green, blue, and red text, respectively. **(B)** PCR analysis to detect the exogenous T-DNA in the T_1_ generation. **(C)** The genotypic segregation ratio of two edited lines in the T_1_ generation and the total number of plants with T-DNA free. The mutation genotypes of three edited lines **(D)** and their plant height were compared with wild type (WT) at seedling **(E)** and maturation stage **(F)**. Scale bar, 2 cm.

The S3 target site was fully matched with a functional copy of *BnaA06.RGA* (*BnaA06g34810D*) which encodes a DELLA protein, serving as a Gibberellins (GA) signaling repressor. A C-to-T substitution conferred a mutation (P91L) in its TVHYNP motif and resulted in a dwarf phenotype (Liu et al., 2010). Sequencing analysis revealed that 108 (41.2%) of the T_0_ plants contained a C-to-T substitution which occurred at C2, C3, C4, C5, C7, and C12 from the protospacer position at the S3 site (Table 1, Supplementary Table 3). A total of 14 different mutation genotypes were detected from the 108 edited lines in the T_0_ generation, and the homozygous substitutions at the conserved P91 in three different lines showed obvious dwarf phenotypes (Figure 5A).

To obtain stable homozygous mutants and test whether the base-editing mutants are inherited, two independent heterozygous lines (BnPBE3-2-8 and BnA3A-PBE-2-86) were self-pollinated. Then, their respective T_1_ progeny were genotyped via Hi-TOM sequencing at the S3 site. In the T_1_ progeny from the BnPBE3-2-8 and BnA3A-PBE-2-86 lines, the segregation ratio observed for the heterozygous, homozygous and wild type genotype was ~1:2:1 (χ^2^ = 0.15 and 0.11, *P* > 0.05; Figure 5C). These results indicated that the produced base substitution was successfully transmitted from T_0_ to T_1_ generation, with an expected monogenic segregation pattern. Furthermore, the PCR assay was performed to detect exogenous T-DNA using the PB-L/R primer pairs (Figure 5B). Twenty edited mutant plants, including seven homozygous mutants without exogenous T-DNA were obtained in the T_1_ generation (Figure 5C).

Indeed, several T_1_ plants with the expected P91L or novel P91F&A92V substitutions showed a decreased plant height compared with wild-type plants (Figures 5D–F). The significant reduction in height was due to a lower first branch position and shorter internodes compared with wild-type plants. Besides, we found that the heterozygous mutants also show a significant reduction in plant height. Previous report showed that the target substitution in *BnaC09.RGA* conserved domain generated dwarf phenotype (Cheng et al., 2020). Altogether, we can conclude that both the functional copies of *BnaA06.RGA* and *BnaC09.RGA* can achieve gain-of-function mutations at the conserved P91 through CBE system. The utilization of these semi-dwarf mutants produced in the study could improve the lodging resistance in rapeseed breeding.

### The Base Editing of the *BnaALS* Gene Produced Herbicide Resistance Rapeseed

**Figure 6:**
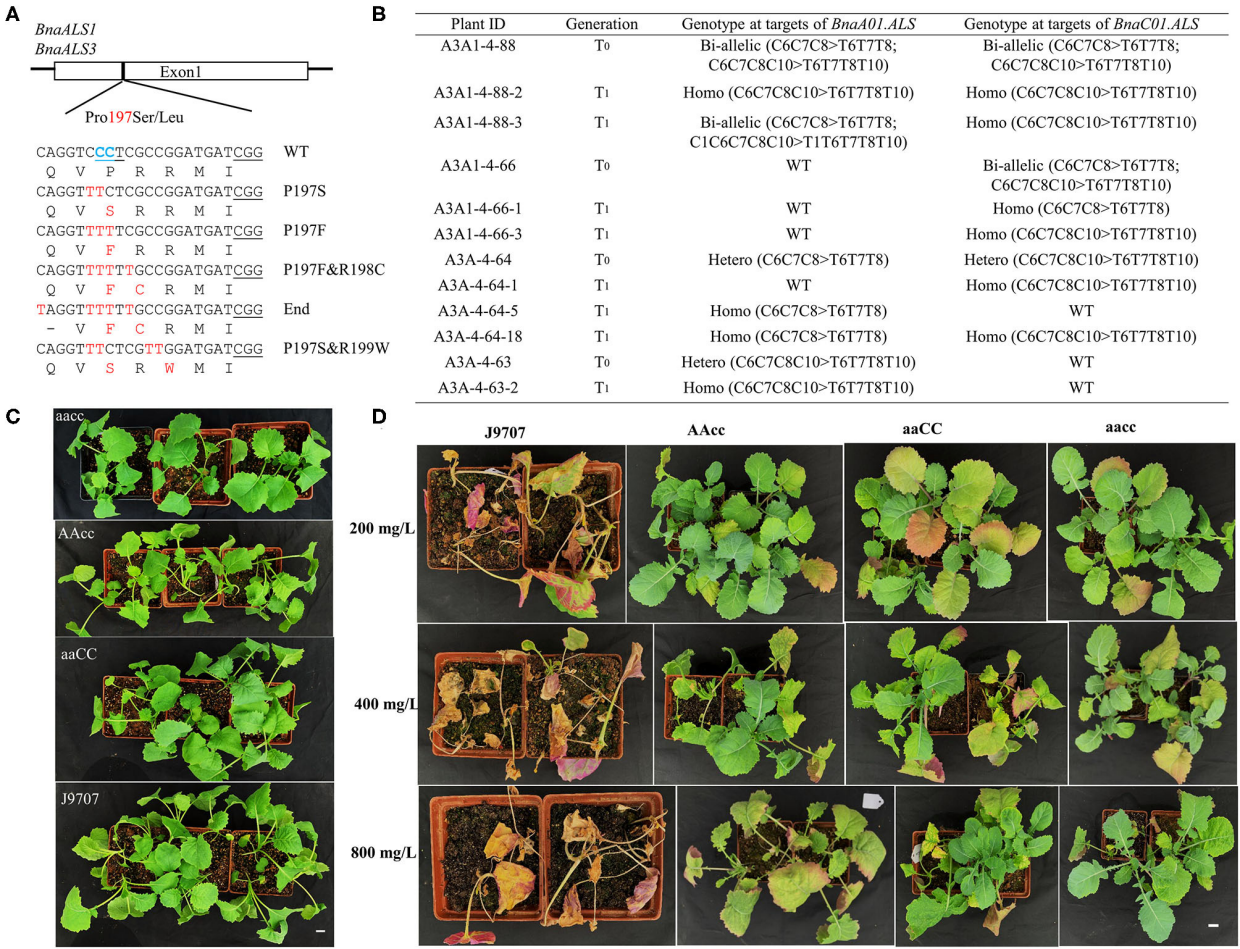
P197F substitution in *BnaALS* T_0_ mutants confer resistance to sulfonylureas herbicides. **(A)** Diverse co-editing events at the P197 site of *BnaALS*. The BnaALS*-*P197 site is conserved in the **(A,C)** subgenomes and 197 was numbered according to the corresponding sequence of Arabidopsis. The PAM were underlined in black line **(B)** Representative P197F substitution in *BnaALS* T_0_ mutants and their transmission to T_1_ generation. Hetero, heterozygous; Homo, homozygous; Bi, biallelic; WT, wild type. **(C,D)** Phenotypes of base-edited plants with different alleles and wild type before and after being tested with 200, 400, and 800 mg/L tribenuron-methyl herbicide in the T_1_ generation. “aaCC,” “AAcc,” and “aacc” represent homozygous mutations of the target gene in *BnaALS3, BnaALS1* and both copies, respectively. Scale bar, 2 cm.

In the edited plants, we were excited to obtain expected substitutions at the conserved P197 site of the acetolactate synthase gene (*BnaALS*) targeted by S10, which probably confer resistance to sulfonylurea herbicides (Li et al., 2015). Sequencing results revealed that 101 (29.0%) of the T_0_ plants contained a C-to-T substitution at the S10 site, which occurred at C1, C6, C7, C8, and C10 from the protospacer position (Table 1). Among these mutant plants, 57 harbored missense mutations in both functional copies of *BnaALS*, among which 21 and 23 had missense mutations in *BnaC01g25380D* (*BnaALS1*) and *BnaA01g20380D* (*BnaALS3*), respectively. Diverse editing events were detected at the target sites of *BnaALS1* and *BnaALS3* (Figure 6A). The expected amino acid substitution (P197S or P197L) was rarely detected in these mutants, whereas most of the editing lines carried P197F and P197F&R198C substitutions (Supplementary Table 3).

To test whether the observed mutations are stably inherited and obtain stable homozygous mutants, four independent T_0_ editing lines of *BnaALS* were self-pollinated to produce T_1_ progeny. The target mutations of progenies from these T_0_ lines were verified by Hi-TOM sequencing analysis of the target sites. As expected, the observed base substitutions were transmitted to the T_1_ generation, and different single and double mutants were obtained (Figure 6B).

To determine whether these P197 missense mutations in the *BnaALS* conferred sulfonylureas herbicide resistance, the T_1_ mutants with homozygous P197-substitutions at a single (AAcc, aaCC) or double (aacc) copies of *BnaALS* gene were treated with various field application levels of tribenuron-methyl at the four-leaf stage. The mutants carrying the P197F edited alleles grew better at 1, 2, and 4 times field-recommended rates (200, 400, and 800 mg/L) over three weeks compared with wild type (Figures 6C,D), the resistance of aacc mutant was the best, followed by AAcc and aaCC mutants at the field application levels of 200 to 800 mg/L sulfonylurea herbicides. Thus, both copies of *BnaALS* gene likely confer herbicide resistance with a similar effect and work in an additive manner (Figure 6D), which is different from the report that *BnaALS3* confer better herbicide resistance than *BnaALS1* (Cheng et al., 2020). Thus, the P197F substitution represents a novel allele which confers herbicide resistance in rapeseed.

### Utilization of Base Editor as a Toolkit for the Insertion of Stop Codon

There are five homologous copies of *BnaIAA7* in the *B. napus* genome, and the S4 was designed to fully target four out of the five gene copies, with *BnaC05.IAA7* having one base mismatched (Supplementary Figure 1A). A G-to-A mutation changed the glycine at the 84th position to glutamic acid (G84E) in *BnaA3.IAA7*, which contributes to reducing the length of internodes and branch angles in rapeseed (Li et al., 2019). As expected, this substitution caused the conversion of the conserved Gly84 to glutamic acid (Supplementary Figure 1B). However, all the edited plants carry a G-to-A mutation at C8, which results in the insertion of a stop codon at the 85th position (Supplementary Figure 1B). Our results suggested that base editing can also be utilized to create knockout mutations by insertion of a stop codon which results in premature termination.

### The NG Protospacer Adjacent Motif Greatly Broaden the Targeting Scope of Base Editing in Rapeseed

In the *B. napus* genome, there are four copies of *BnaDA1* gene. S5 was designed to target both *BnaA06.DA1* and *BnaC08.DA1*, while S6 and S7 were designed to target *BnaC05.DA1* and *BnaA08.DA1*, respectively. We designed these targets intending to obtain base substitutions at the conserved Arg358 of the *BnaDA1* (DA means big in Chinese) targeted by S5–S7. The A358K conversion of *BnaDA1* probably contributes to the improvement of seed weight in rapeseed (Wang et al., 2017). S8 and S9 were designed to target *BnaALS3* and *BnaALS1*, respectively. The alanine at position 122 of *ALS* is converted to valine, which endow mutant resistance to imidazolinone herbicide (Li et al., 2008; Sala et al., 2008; Han et al., 2012). In the T_0_ edited plants, only 11 edited plants contained the intended R358K conversion at S5–S7 and only 1 edited plant contained the intended A122V conversion at S8–S9 since the target bases (C13 or C15) are located outside of the hot spot of the deamination window (Figure 3; Supplementary Table 5). Thus, it is imperative to develop an engineered SpCas9 variant that recognizes not only the NGG protospacer adjacent motif (PAM), to ensure that the desired target bases are located within the hot spot of the editing window. The seed weight of the 11 edited plants containing the intended R358K conversion at S5–S7 will be tested in the next generation since there were limited seeds to conduct a field experiment in the T_1_ plants.

**Figure 7:**
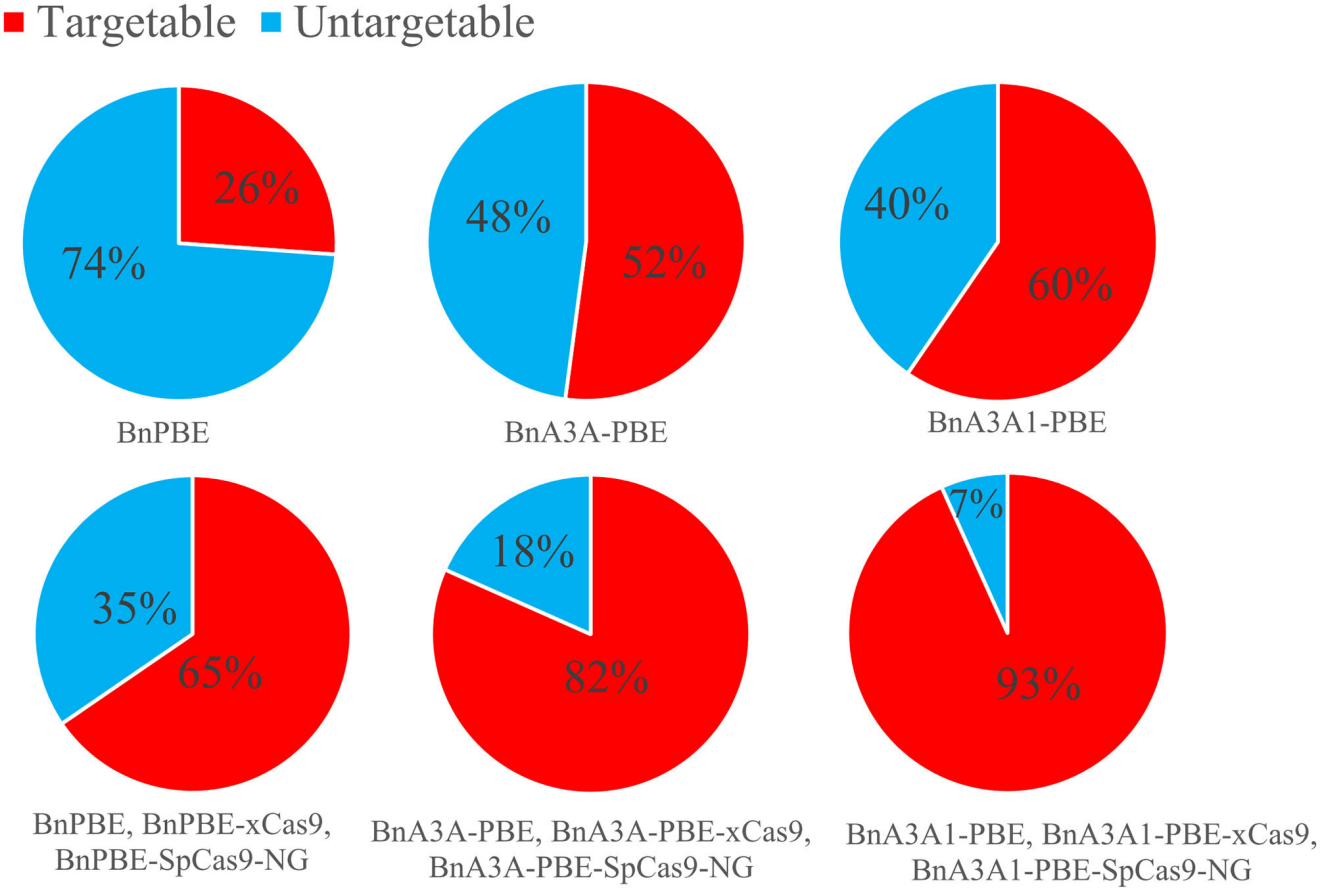
Computational analysis of the rapeseed genome revealed that Cytidines (NGG PAM) and guanidines (CCN PAM) can potentially be edited by BnPBE, BnA3A-PBE, and BnA3A1-PBE. The above three CBEs together with the various Cas9 variants (SpCas9 and xCas9) promise to increase the scope of base editing of targeted cytidines and guanidines in rapeseed genome.

*Streptococcus pyogenes* Cas9 (SpCas9) recognizes a very simple NGG PAM, making it the most commonly used CRISPR-Cas9 system. The canonical NGG PAM limits its targeting scope in a genome, especially for applications that require precise Cas9 positioning such as base editing (Wang et al., 2019). Recently, the engineered SpCas9 variant, SpCas9-NG, which recognize NG PAMs are more efficient than the xCas9 variant (Hu et al., 2018; Nishimasu et al., 2018), Moreover, SpCas9-NG coupled with the activation-induced cytidine deaminase (AID) can mediate the C-to-T conversion at target sites with NG PAMs in human cells (Nishimasu et al., 2018). Endo et al. (2019) reported that the SpCas9-NG can efficiently mutagenize endogenous target sites with NG PAMs in rice and Arabidopsis genomes. For *B. napus* genome, the 17-nt editing window of BnA3A1-PBE theoretically increases up to 15.3% and 1.3-fold the number of genomic cytidines and guanidine available for base editing when compared to BnA3A-PBE and BnPBE, respectively (Figure 7). When combined with SpCas9, xCas9, and other variants with NG PAM, BnA3A1-PBE theoretically targets 93% of the cytidines and guanidine in the rapeseed genome (Figure 7), which makes it as an ideal editing system for further improvement in future research.

In conclusion, the three CBEs described here can efficiently and specifically perform precise C-to-T substitutions across a broad range of endogenous genomic loci in rapeseed. The improved BnA3A1-PBE performed efficiently as a base editor with higher editing efficiency, a more broadened editing window coupled with a higher proportion of homozygous and biallelic genotypes compared with BnA3A-PBE and BnPBE systems. When compared with the two latest studies which reported the successful application of cytosine base-editing in rapeseed (Wu et al., 2020, Cheng et al., 2020), BnA3A1-PBE has a high editing efficiency, which is ~16-fold than PBE (Wu et al., 2020), and 26% higher than those of A3A-PBE (Cheng et al., 2020). Thus, BnA3A1-PBE represents a powerful base editor for both gene function study and molecular breeding in rapeseed.

## Data Availability Statement

Sequencing data has been deposited in NCBI Sequence Read Archive (SRA) database under the accession no. PRJNA663415.

## Author Contributions

LH, CF, and YZho: conceived and designed the experiments. LH, QL, OA, SC, MZ, XS, KY, YZha, YY, and LX: performed the experiments. LH and CF: wrote the manuscript. All authors contributed to the article and approved the submitted version.

## Conflict of Interest

The authors declare that the research was conducted in the absence of any commercial or financial relationships that could be construed as a potential conflict of interest.

## Supplementary Material

Supplementary Figure 1 The desired substitution accompany with a stop codon occurred at the S4 target site. **(A)** The target sequences are shown with the PAM underlined in red line. **(B)** Diverse co-editing events at the BnaIAA7*-*Gly84 site, all of these new alleles introduce an early stop codon.

Supplementary Table 1 The primers used in this study.

Supplementary Table 2 Detail information on the numbers of T_0_ plants in BnPBG14 system.

Supplementary Table 3 Detail information of the mutation of all edited T_0_ plants in this study.

Supplementary Table 4 Summary of deep sequencing data for most likely off-target sites of S1, S2, and S3.

Supplementary Table 5 The edited lines contain the intended C13 to T13 conversion at S5-S7.
