# Supplementary figures and images for: Precision Genome Engineering Through Cytidine Base Editing in Rapeseed (Brassica napus. L)
Source: Front Genome Ed. 2020 Nov 20;2:605768. doi: 10.3389/fgeed.2020.605768 (PMC8525351; doi:10.3389/fgeed.2020.605768)

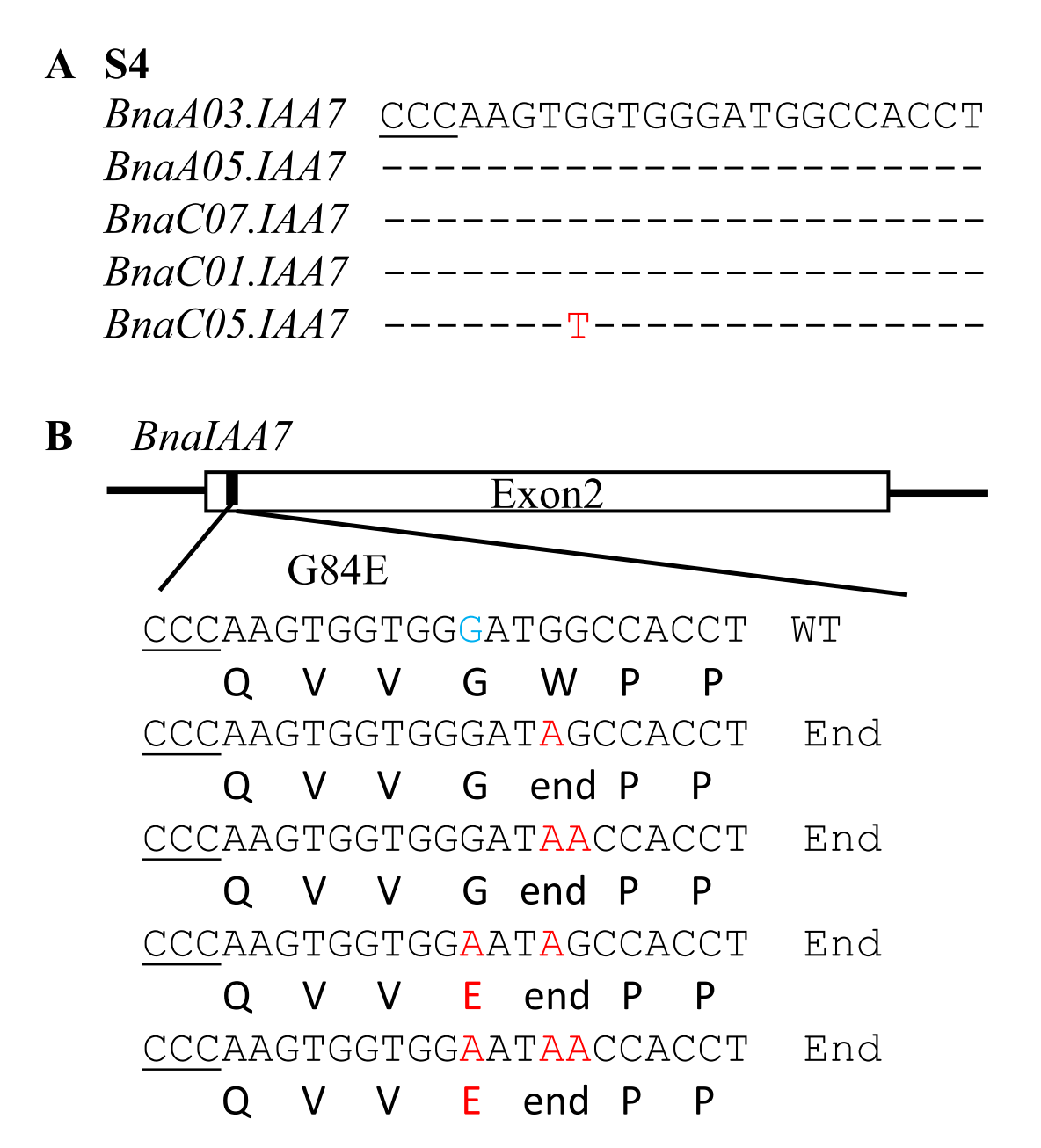

Supplement: Supplementary Figure 1 — The desired substitution accompany with a stop codon occurred at the S4 target site. (A) The target sequences are shown with the PAM underlined in red line. (B) Diverse co-editing events at the BnaIAA7-Gly84 site, all of these new alleles introduce an early stop codon. [file Image_1.TIF]
